# Modulating the *T*_g_ of Poly(alkylene succinate)s by Inserting Bio-Based Aromatic Units via Ring-Opening Copolymerization

**DOI:** 10.3390/polym9120701

**Published:** 2017-12-12

**Authors:** Juan Carlos Morales-Huerta, Antxon Martínez de Ilarduya, Sebastián Muñoz-Guerra

**Affiliations:** Departament d’Enginyeria Química, Universitat Politècnica de Catalunya, ETSEIB, Diagonal 647, 08028 Barcelona, Spain; juan.carlos.morales.huerta@upc.edu (J.C.M.-H.); antxon.martinez.de.ilarduia@upc.edu (A.M.d.I.)

**Keywords:** bio-based copolyesters, poly(alkylene succinate)s, resorcinol, 2,5-furandicarboxylic acid, ED-ROP, furane-based polyesters

## Abstract

Two series of aliphatic-aromatic copolyesters derived from succinic and 2,5-furandicarboxylic acids, and di-*O*-2-(hydroxyethyl) resorcinol as diol substituent of either 1,4-butanediol or ethylene glycol, respectively, were obtained by ring opening polymerization(ROP) performed in bulk and catalyzed by Sn(Oct)_2_. Cyclic oligomers of furandicarboxylate of di-*O*-2-(hydroxyethyl) resorcinol were successfully synthesized by high-dilution condensation, and then copolymerized with cyclic oligomers of either butylene or ethylene succinate. The synthesized resorcinol-containing succinate-furanoatecopolyesters had *M*_w_ oscillating between 50,000 and 30,000 g·mol^−1^ depending on composition, and they all displayed a nearly random microstructure. They showed an excellent thermal stability with onset decomposition temperatures near 300 °C. They are amorphous with *T*_g_ increasing monotonically with the content in resorcinol in both series with values ranging from −30 or −13 °C for butylene and ethylene-based copolyesters, respectively, up to around 45 °C. The resorcinol-containing succinate-furanoate copolyesters showed appreciable hydrolytic degradation when incubated for a few weeks in water under physiological conditions, a behavior that was notably enhanced in the presence of lipases.

## 1. Introduction

Poly(alkylene succinate)s area family of aliphatic polyesters that are distinguished by their easy accessibility from naturally occurring compounds and their noticeable biodegradability [1,2]. In fact, the US Department of Energy declared bio-based succinic acid as a high-potential chemical platform for compounds so far obtained from fossil feedstock, which is indeed the case of poly(succinate)s [3]. Also most alkanediols, and in particular 1,2-ethanediol and 1,4-butanediol, are compounds accessible from renewable resources [4,5,6]. Poly(alkylene succinate)s are highly crystalline polyesters with thermal and mechanical properties largely depending on the length of the alkylene moiety [7,8]. Poly(butylene succinate) (PBS) and poly(ethylene succinate) (PES), with melting temperatures of 115 and 100 °C respectively, are by far the most well-known members of the family, the former being currently produced at industrial scale, and both of them showing excellent potential for packaging and biomedical applications [9]. Nonetheless, due to the high flexibility inherent to the aliphatic polyester chain, the glass transition temperatures of poly(alkylene succinate)s are too low, with values around −30 and −12 °C for PBS and PES, respectively. Copolymerization including benzenedicarboxylic acids as comonomers has proven to be a highly effective method to increase the *T*_g_ of the aliphatic polyesters [10]. This is not however a fully satisfactory approach since the sustainability of the parent aliphatic polyester becomes largely reduced, and its biodegradability is almost lost when significant amounts of aromatic moieties are introduced in the polymer chain [11]. Copolymerization using bio-based 2,5-furandicarboxylic acid has represented a noteworthy step forward in this regard. Poly(butylene succinate-*co*-2,5-furandicarboxylate) copolyesters, abbreviated as *co*(PBS_x_BF_y_), with *T*_g_ within the ±30 °C range depending on composition, and retaining appreciable biodegradability, have been described by different authors [12,13,14]. Also Bikiaris et al. have recently reported on poly(ethylene succinate-*co*-2,5-furandicarboxylate)s, abbreviated as *co*(PES_x_EF_y_), which display *T*_g_ increasing monotonically up to near 80 °C and whichare able to show perceptible biodegradability even for high contents in furanoate units [15].

In the present work, copolyesters of PES and PBS containing both 2,5-furandicarboxylate (F) and 1,3-benzenedioxyethylene (R) units, which derive from the 2,5-furandicarboxilic acid (FDCA) and resorcinol, respectively, are reported. FDCA is a well-known bio-based compound that is currently receiving enormous attention as a possible substitute of terephthalic and isophthalic acids in the industrial synthesis of aromatic polyesters [16,17,18]. Resorcinol (1,3-dihydroxybenzene) is a diphenol currently produced at industrial scale by alkaline fusion of 1,3-benzenedisulfonic acid but that may be also obtained from glucose by fermentation either via inositol [19,20] or via triacetic acid lactone [21]. This compound is extensively used in medicine as external antiseptic [22], and it is also consumed in large amounts in the industrial manufacture of formaldehyde resins [23].

The synthesis of linear polyesters using a resorcinol-based monomer was firstly reported by Vijayakumar et al. [24] and later by Gioia et al. [25]. These authors etherified resorcinol with ethylene carbonate todi-*O*-2-(hydroxyethyl) resorcinol (HER). This primary aliphatic diol allowed the avoidance ofthe polymerization restraints derived from the low reactivity of the aromatic hydroxyl groups. HER was made to react with both aromatic and aliphatic dicarboxylic acids, both saturated and unsaturated, to obtain an assortment of potentially sustainable amorphous polyesters. Resorcinol-containing polymers with *M*_w_ up to near 55,000 g·mol^−1^ were reported by Goia et al. [25]. Additionally, several patents covering diverse aromatic copolyesters of resorcinol displaying high barrier properties have been also reported [26,27]. The ability of resorcinol to repress gas permeability in polymers is in line with the general behavior that is characteristic of 1,3-disubstituted aromatic compounds.

The aim of the present paper was to obtain PES and PBS copolyesters with enhanced *T*_g_ but without losing the potential sustainability of the parent homopolyesters. The hypothesis applied to the work was that the substitution of the alkylene unit by a resorcinol-derived unit in poly(alkylene succinate-*co*-furanoate) copolyesters may further increase the stiffness of the chain in a similar manner as aromatic diacids do. In order to favor the sustainability of the synthesis, the copolyesters have been prepared by ring-opening copolymerization in bulk following the methodology previously used by us for the synthesis of *co*(PBS_x_BF_y_) copolyesters [14]. By this method solvents are avoided and generation of byproducts is minimized. Cyclic oligomers of furandicarboxylate of di-*O*-2-(hydroxyethyl) resorcinol required for ring opening polymerization (ROP) have been prepared for the first time and characterized following the same methodology reported by us for the synthesis of butylene furanoate oligomers. By this means, two series of succinate-furanoatecopolyesters, made from mixtures of either 1,4-butanediol or ethylene glycol, and di-*O*-2-(hydroxyethyl) resorcinol, have been obtained for a wide range of compositions. The thermal behavior and biodegradability of the two copolyesters series was comparatively evaluated between them and also taking as reference the poly(alkylene succinate-*co*-furanoate) series reported in the recent literature [14,15].

## 2. Materials and Methods

### 2.1. Materials

2,5-Furandicarboxylic acid (FDCA, 98% purity) was purchased from Satachem (Shanghai, China). Di-*O*-2-(hydroxyethyl) resorcinol (HER) (99%) was obtained from TCI Chemicals (Tokyo, Japan), thionyl chloride (SOCl_2_, 99%), 1,4-butanediol (BD, 99%), ethylene glycol (EG, 99%), di-azabicyclo[2.2.2]octane (DABCO, 99%), tin(II) ethylhexanoate (Sn(Oct)_2_, 99%) catalysts, and porcine pancreas lipase were purchased from Sigma-Aldrich Co. (St. Louis, MO, USA).Triethylamine (Et_3_N, 98%) and dimethyl succinate (DMS) were purchased from Panreac (Castellar del Vallés, Spain). Solvents used for reaction, isolation and purification were of high-purity grade and used as received, except dichloromethane (DCM), tetrahydrofurane (THF) and toluene that were dried on 3 Å-molecular sieves. DABCO catalyst was purified by sublimation.

### 2.2. Measurements

^1^H and ^13^C NMR spectra were recorded on a Bruker AMX-300 spectrometer (Bruker, Billerica, MA‎, USA) at 25 °C, operating at 300.1 and 75.5 MHz, respectively. For NMR analysis, monomers, cyclic oligomers, intermediate compounds, and polymers were dissolved in deuterated chloroform (CDCl_3_), and spectra were internally referenced to tetramethylsilane (TMS). About 10 and 50 mg of sample in 1 mL of solvent were used for ^1^H and ^13^C NMR, respectively. Sixty-four scans were recorded for ^1^H and between 1000 and 10,000 scans for ^13^C NMR.

High-performance liquid chromatography (HPLC) analysis was performed at 25 °C in a Waters apparatus equipped with a UV detector of Applied Biosystems(Foster City, CA, USA) operating at 254 nm wavelength, and a Scharlau Science column (Si60, 5 μm; 250 × 4.6 mm, Scharlab, Sentmenat, Spain). Cyclic oligomers (1 mg) were dissolved in chloroform (1 mL) and eluted with hexane/1,4-dioxane 70/30 (*v*/*v*) at a flow rate of 1.0 mL·min^−1^.

Matrix-assisted laser desorption/ionization time of flight (MALDI-TOF) mass spectra were recorded in a 4700 Proteomics Analyzer instrument (Applied Biosystems, Foster City, CA, USA) of the Proteomics Platform of Barcelona Science Park, University of Barcelona. Spectra acquisition was performed in the MS reflector positive-ion mode. About 0.1 mg of sample was dissolved in 50 μL of DCM and 2 μL of this solution were mixed with an equal volume of DCM solution of anthracene (10 mg·mL^−1^) and this mixture left to evaporate to dryness onto the stainless steel plate of the analyzer. The residue was then covered with 2 μL of a solution of 2,5-dihydroxibenzoic acid in acetonitrile/H_2_O (1/1) containing 0.1% trifluoroacetic acid (TFA), and the mixture was left to dry prior to exposition to the laser beam.

Molecular weight analysis was performedby GPC on a Waters equipment provided with RI and UV detectors. 100 μL of 0.1% (*w*/*v*) sample solution were injected and chromatographed with a flow of 0.5 mL·min^−1^ of 1,1,1,3,3,3-hexafluoroisopropanol (HFIP). HR5E and HR2 Waters linear Styragel columns (7.8 mm × 300 mm, pore size 103–104 Å) packed with crosslinked polystyrene and protected with a pre-column were used. Average molecular weights and molecular dispersities were calculated against PMMA standards. 

The thermal behavior of cyclic compounds and polymers were examined by differential scanning calorimetry (DSC), using a (Perking-Elmer Pyris apparatus, Waltam, MA, USA). Thermograms were recorded on 3–6 mg samples in the −80–200 °C interval at heating or cooling rates of 10 °C·min^−1^ under a nitrogen flow of 20 mL·min^−1^. Indium and zinc were used as standards for temperature and enthalpy calibration, respectively. The glass transition temperature (*T*_g_) was taken as the inflection point of the heating DSC traces recorded at 20 °C·min^−1^ from quenched samples, and the melting temperature (*T*_m_) was taken as the maximum of the endothermic peak appearing on heating traces. Thermogravimetric analysis was performed on a Mettler-Toledo TGA/DSC 1 Star System under a nitrogen flow of 20 mL·min^−1^ at a heating rate of 10 °C·min^−1^ and within a temperature range of 30 to 600 °C.

### 2.3. Synthesis of Cyclic Oligomers

Cyclic oligomers of butylene succinate and ethylene succinate, *c*(BS)_n_ and *c*(ES)_n_ respectively, were prepared by the high-dilution condensation (HDC) method [28] following the experimental procedures reported by us [14,29]. Cyclic oligo[di-*O*-2-(hydroxyethyl) resorcinol 2,5-furandicarboxylate]s, abbreviated as *c*(RF)_n_, were synthesized from HER and 2,5-furandicarboxylic dichloride (FDCA-Cl_2_) by applying the HDC methodology in a manner as we reportedfor the synthesisof cyclic oligo(butylene 2,5-furandicarboxylate)s with some minor modifications [30]. To 250 mL of THF placed in a three-necked round bottom flask at 0 °C, 6.30 mmol (0.71 g) of DABCO were added under stirring. 2.55 mmol (0.48 g) of FDCA-Cl_2_ and 2.55 mmol (0.5 g) of HER in 10 mL of THF were then drop-wise added simultaneously over a period of time of 40 min using separated addition funnels in order to maintain the reagents equimolarity. The reaction was finished by adding 1 mL of water followed by 5 mL of 1 M HCl. After stirring for 5 min, the reaction mixture was diluted with DCM and filtered. The filtrate was washed with 0.1 M HCl, dried on MgSO_4_, and evaporated to dryness to render a mixture of linear and cyclic oligomers. Linear oligomers were removed by chromatography through a short column of silica gel using a cold mixture of DCM/diethyl ether 90/10 (*v*/*v*) as eluent. Yield: 60%. ^1^H NMR (δ ppm, CDCl_3_, 300 MHz): 7.30–7.10 (m), 6.63 (dd), 6.54 (m), 4.67 (m), 4.56 (m), 4.46 (m), 4.26 (m). ^13^C NMR (δ ppm, CDCl_3_, 75.5 MHz): 161.6, 159.6, 157.8, 146.5, 130.0, 129.8, 118.9, 118.4, 111.7, 107.4, 107.2, 102.1, 69.2, 65.7, 65.6, 64.9, 63.7, 63.6.

### 2.4. Synthesis of Polyesters

Polyesters and copolyesters were synthesized by ROP in bulk using Sn(Oct)_2_ as catalyst. The general procedure was as follows: the mixture made of *c*(RF)_n_ and either *c*(BS)_n_ or *c*(ES)_n_ at the definite molar ratio and containing 1 mol % of catalyst, was dissolved in CHCl_3_ in a three-necked round bottom flask, and then evaporated to dryness and held under vacuum for 24 h. Reaction was then initiated by heating the mixture at the selected temperature and left it to proceed for the scheduled time under mechanical stirring and under a nitrogen flow. Aliquots were removed at scheduled times and analyzed by GPC in order to follow the evolution of molecular weight as the reaction proceeded.

### 2.5. Hydrolytic and Enzymatic Degradation

Copolyesters *co*PBS_40_RF_60_, *co*PBS_50_RF_50_, *co*PBS_70_RF_30_, *co*PES_40_RF_60_, *co*PES_50_RF_50_ and *co*PES_70_RF_30_ as well as the parent homopolyesters PBS, PES and PRF were selected for the degradation study. Films with a thickness of ~200 μm were prepared by casting from either HFIP or chloroform solution of the polymer at a concentration of 100 g·L^−1^. Films were cut into 10 mm-diameter, 20–30 mg-weight disks and dried under vacuum to constant weight. For hydrolytic degradation assays, samples were immersed in vials containing 10 mL of sodium phosphate buffer, pH 7.4 at 37 °C. Enzymatic degradation assays were carried out in the same buffer but with 10 mg of porcine pancreas lipase added. In this case, the buffer solution was replaced every 72 h in order to insurance enzyme activity. In both cases, disks were withdrawn from the incubation medium at scheduled times, washed carefully with distilled water, dried to constant weight, and analyzed by GPC.

## 3. Results and Discussion

**Scheme 1:**
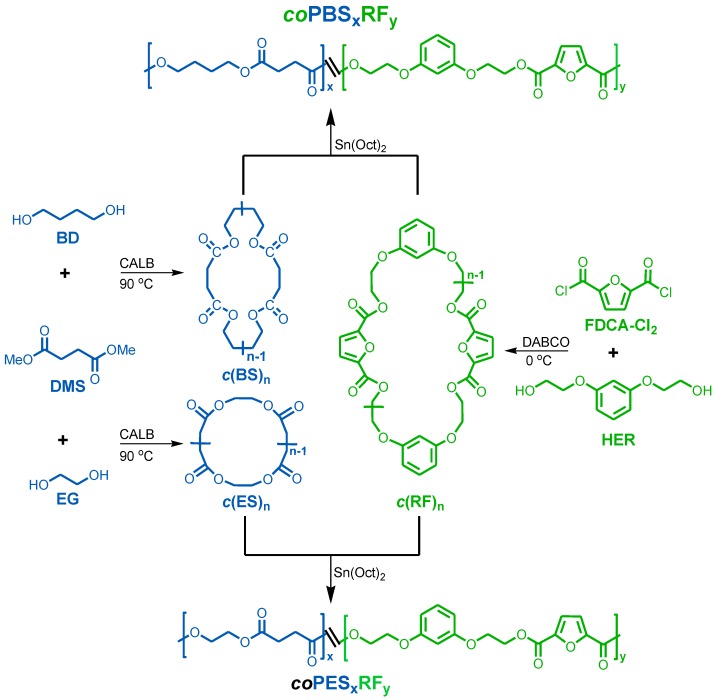
Route of synthesis leading to PBSRF and PESRF copolyesters.

The synthetic route followed in this work for the preparation of *co*PBS_x_RF_y_ and *co*PES_x_RF_y_ copolyesters by ROP including the synthesis of the cyclic oligomers and their polymerization is depicted in Scheme 1.

### 3.1. Synthesis of Cyclic Oligomers

The cyclic oligomers *c*(BS)_n_ and *c*(ES)_n_ were first synthesized by enzymatic cyclocondensation in solution of dimethyl succinate with either 1,4-butanediol or ethylene glycol, respectively, applying the general procedure previously described by us [30]. A summary of relevant features of these cyclic oligomers fractions is given in Table 1. It is worth noticing that both fractions are heterogeneous in cycle sizes and that no dimer is detected in the *c*(ES)_n_ fraction.

**Figure 1:**
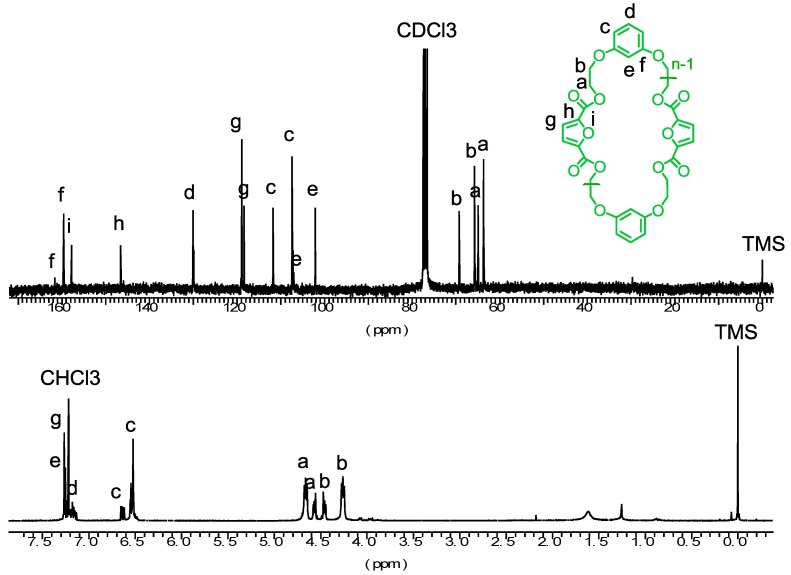
^1^H and ^13^C NMR spectra of *c*(RF)_n_.

The *c*(RF)_n_ fraction has been synthesized for the first time in this work from FDCA-Cl_2_ and HER in 60% yield after removing the linear oligomers by flash chromatography. The ^1^H and ^13^C NMR spectra of this purified oligomeric fraction were fully consistent with the expected cyclic chemical structure (Figure 1). Splitting displayed by some signals is due to magnetic environment differences between protons and carbons attached to the cyclic dimer and to other cyclic oligomers of higher size, which become distinguishable by NMR.

**Figure 2:**
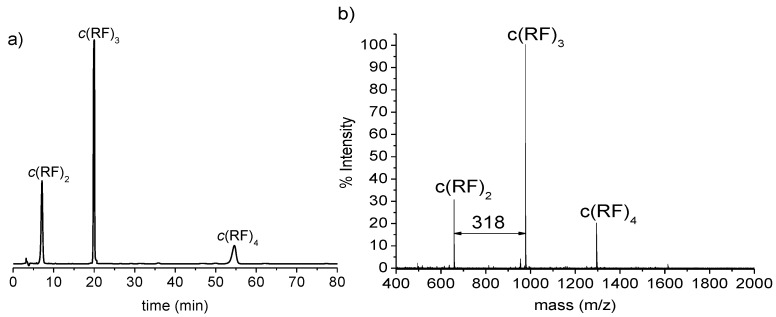
HPLC (**a**) and MALDI-TOF (**b**) of *c*(RF)_n_.

The composition of the *c*(RF)_n_ fraction was determined by HPLC chromatography and MALDI-TOF spectroscopy, and results obtained therefrom are shown in Figure 2. Three peaks were observed in the HPLC chromatograms which correspond to dimer, trimer and tetramer species in an approximate molar ratio of 2/4/1, as it was ascertained in the MALDI-TOF spectra. According to these results, the content of this fraction in species of size higher than tetramer may be considered almost negligible.

**Figure 3:**
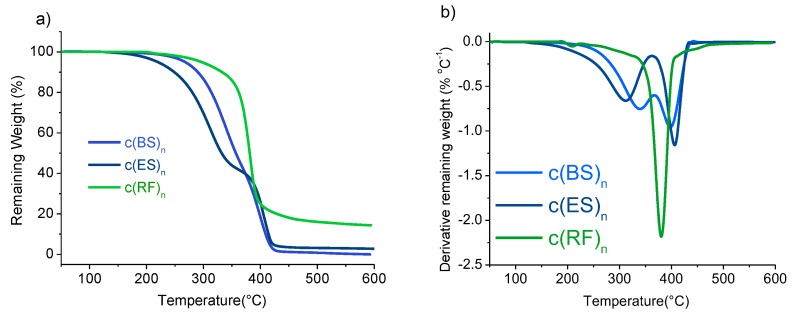
Compared TGA analysis of cyclic oligomers. (**a**) TGA traces; (**b**) Derivative curves showing temperatures at which degradation takes place at maximum rate.

As it is seen in Table 1, thermal properties of *c*(RF)_n_ differ significantly from those of *c*(BS)_n_ and *c*(ES)_n_. These resorcinol-containing cycles not only melt at higher temperature but also display multiple peaks probably arising from cyclic species of different size. On the other hand, *c*(RF)_n_ shows much greater resistance to decomposition by heating than the alkylene succinate cycles, as it should be expected from their aromatic constitution. Furthermore, as it is shown in Figure 3, the TGA trace of *c*(RF)_n_ only shows one single weight loss step at difference with the two well separate falls that are observed for alkylene succinate cycles. This is a very reasonable result since the weight losses displayed by *c*(BS)_n_ and *c*(ES)_n_ at temperatures in the 300–350 °C range are reported to be due to partial volatilization of the sample, a fact that is not expected to happen in the case of *c*(RF)_n_. 

### 3.2. Synthesis of Copolyesters 

**Figure 4:**
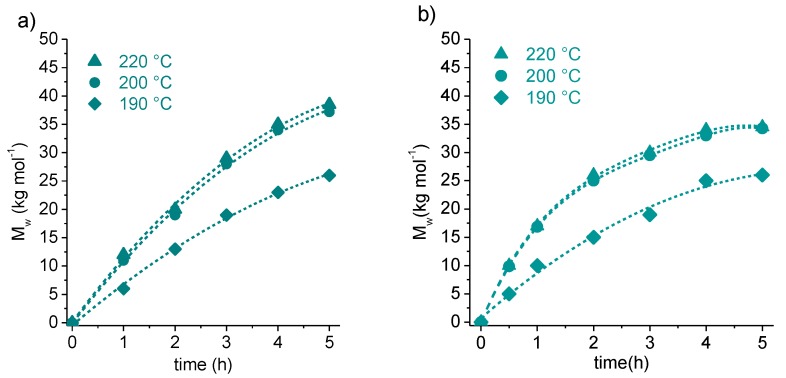
Evolution of*M*_w_along reaction time in the ROP of equimolar mixtures of *c*(RF)_n_ and either *c*(BS)_n_ (**a**) or *c*(ES)_n_ (**b**) forthree different temperatures.

**Figure 5:**
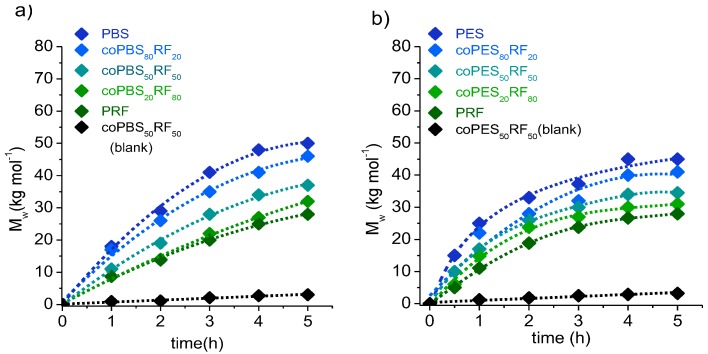
Evolution of *M*_w_ along reaction time in the ROP of mixtures of *c*(RF)_n_ and either *c*(BS)_n_ (**a**) or *c*(ES)_n_ (**b**) for different feed compositions.

The synthesis of the copolyesters was performed by ED-ROP (Entropically-Driven Ring Opening Polymerization) in bulk using Sn(Oct)_2_ as catalyst. Two copolyesters series, i.e., *co*PBS_x_RF_y_ and *co*PES_x_RF_y_, were prepared frommixtures of either *c*(BS)_n_ or *c*(ES)_n_ and *c*(RF)_n_ at molar ratios ranging between 10% and 90%, in addition to the homopolyesters PBS, PES and PRF. Reaction temperatures above 190 °C were used to insure melting of the cycles. Preliminary assays indicated that reaction noticeably speeded when temperature was raised from 190 to 200 °C but not significant change in rate was observed when temperature was further increased, a behavior that was shared by the two series (Figure 4). According to these results, this was the temperature of choice applied for the synthesis of the whole collection of copolyesters. The evolution of the molecular weight of the forming polymer as a function of reaction time for several selected compositions is represented for the two series in Figure 5. An asymptotic trend was followed in all cases showing a notable decay in reaction rate after five hours when weight-average molecular weight values up to ~40,000 and ~50,000 g·mol^−1^ for PBS and PES copolyesters, respectively, were attained. Lower values, around 28,000–30,000 g·mol^−1^ were indeed achieved when higher contents of RF were used.

Interestingly, ROP proceeded faster as the content of the feed in *c*(RF)_n_ diminished, which may be interpreted as due to the higher relative reactivity of the alkylene succinate cycles. An assay of polymerization in absence of catalyst showed not significant increase in molecular weight indicating that ROP was unable to proceed by exclusive action of heating.

**Figure 6:**
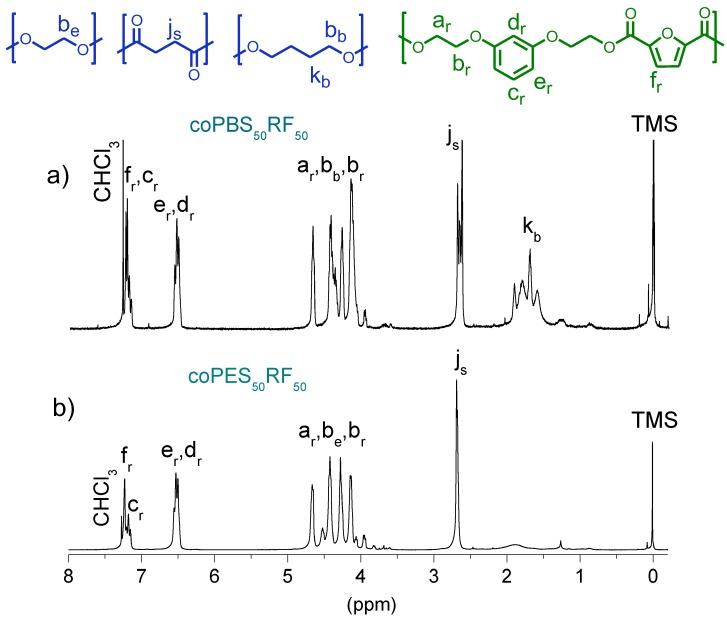
^1^H NMR spectra of resorcinol-containing succinate-furanoatecopolyesters. (**a**) *co*PBS_50_RF_50_; (**b**) *co*PES_50_RF_50_.

The NMR analysis of the copolyesters ascertained the chemical constitution expected for them. ^1^H NMR spectra of *co*PBS_50_RF_50_ and *co*PES_50_RF_50_ are shown in Figure 6 as representative examples and the whole spectra collection for the two series is accessible in the SI file (Figures S1 and S2). The comonomer content in the copolyesters determined from the information provided by ^1^H NMR spectra revealed a good correlation with the composition used in the feed with deviations being always below 5%. The values obtained for every case are detailed in Table 2. 

### 3.3. Microstructure of the Copolyesters

The number average sequence lengths (*n*) present in the copolyesters as well as their degrees of randomness (*B*) were determined by NMR. For this analysis we have applied the statistical model described by Devaux et al. [31] for polycondensates

[S] = [B] or [S] = [E], and [F] = [R] and [BF] = [RS] or [EF] = [RS], for the two series. 

**Figure 7:**
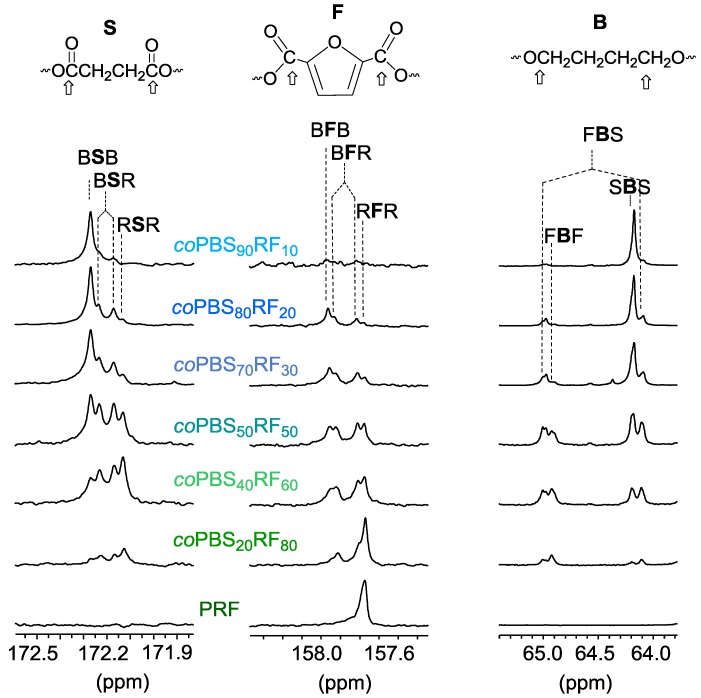
Aromatic and aliphatic carbonyl carbons regions of the ^13^C NMR spectra of *co*PBS_x_RF_y_ copolyesters used for calculation of their microstructure.

The number average sequence lengths (*n*) and the degree of randomness (*B*) were calculated using the following expressions:

$$n_{BS}=\frac{\left[BS\right]}{\left[BF\right]}+1,\;n_{BF}=\frac{\left[BF\right]}{\left[BS\right]}+1,\;n_{RS}=\frac{\left[RS\right]}{\left[RF\right]}+1,\;n_{RF}=\frac{\left[RF\right]}{\left[RS\right]}+1$$

$$B=\frac{1}{n_{BS}}+\frac{1}{n_{RF}}$$

$$n_{ES}=\frac{\left[ES\right]}{\left[EF\right]}+1,\;n_{EF}=\frac{\left[EF\right]}{\left[ES\right]}+1,\;n_{RS}=\frac{\left[RS\right]}{\left[RF\right]}+1,\;n_{RF}=\frac{\left[RF\right]}{\left[RS\right]}+1$$

$$B=\frac{1}{n_{ES}}+\frac{1}{n_{RF}}$$

where the dyads fractions were calculated from the integrals of the ^13^C NMR spectra, and assuming similar relaxation times for the same carbons in the different dyads. It was additionally assumed that in all triads the substitution on the left of a central unit does not influence the substitution on the right. The ^13^C NMR spectra of the *co*PBS_x_RF_y_ series are shown in Figure 7 where it is seen that some of the signals split into four peaks due to the sensitivity of these carbons to sequence distribution at the level of triads. By deconvolution of these signals the content of the different dyads could be easily obtained and then *n*_BS_ and *n*_RF_, and the degree of randomness *B* calculated by applying the equations described above. A similar approach was used for the analysis of the *co*PES_x_RF_y_ series whose ^13^C NMR spectra are shown in Figure S3 of the SI file. *B* an *n* values for the two series are given in Table 2 revealing that the microstructure of the two series of copolyesters is essentially at random.

Results obtained in this microstructural analysis bring into evidence that transesterification reactions must happen during polymerization. In the absence of such reactions, average lengths for *n*_(B/E)S_ and *n*_RF_ homosequencesare expected to be higher than 2 for any composition. The values below 2 frequently observed in the two series must be consequence of the existence of transreactions leading to randomization. An unquestionable proof of the occurrence of transesterifications is the detection by ^13^C NMR of RSR, BFB or FBF sequences, which would not be allowed if only ROP reactions take place. The molecular dispersities displayed by the obtained copolyesters, which are between 1.4 and 2.0, are affected by such transesterification reactions. This is expected for ED-ROP because reactions of the active ends of the linear growing chains with the ester linkages of other chains and with the ester groups of the strainless macrolactone occur at similar rates. Accordingly, dispersities of polymeric products from ED-ROP will be about 2 in the case that a full equilibration is reached [32].

### 3.4. Thermal Properties

The thermal properties of the *co*PBS_x_RF_y_ and *co*PES_x_RF_y_ copolyesters were evaluated by TGA and DSC, and the most relevant data provided by these analyses are collected in Table 3. 

**Figure 8:**
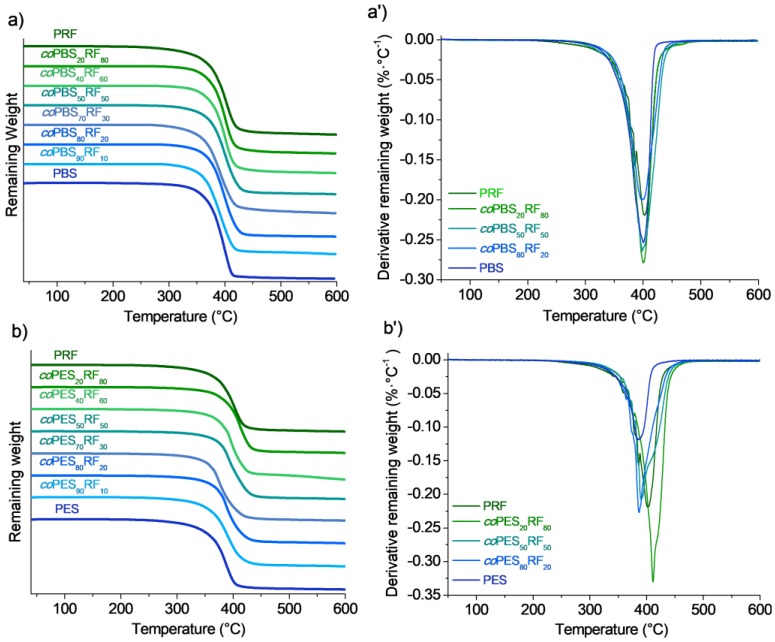
TGA traces of *co*PBS_x_RF_y_ and *co*PES_x_RF_y_ series (**a**,**b**) and their derivative curves (**a’**,**b’**).

The TGA traces of all copolyesters recorded under a nitrogen atmosphere, as well as their derivative curves, are shown in Figure 8. The main conclusion drawn from TGA results is that succinate-furanoate copolyesters containing resorcinol are well stable to non-oxidative heating. In fact, the onset temperature is always above 320 °C and maximum decomposition rate temperatures are not far from 400 °C. Slight differences observed between the two series should be related to the intrinsically lower thermal stability of PES as compared to PBS. As it is well illustrated by the derivative curves shown in Figure 8a’,b’, the decomposition process takes place essentially in a single stage although a smooth shoulder leans out some *co*PES_x_RF_y_ peaks indicating a greater complexity in the thermal decomposition of this series.

The DSC traces of the *co*PBS_x_RF_y_ and *co*PES_x_RF_y_ copolyesters did not show any heat exchange indicative of melting except for *co*PBS_90_RF_10_ that displayed an endothermal peak at 100 °C with an associate enthalpy of 74 J·mol^−1^. This behaviour contrasts with that of both PBS and PES homopolyesters which are highly crystalline, but is in agreement with the amorphous character of PRF. It is apparent that the insertion of the RF units in the poly(alkylene succinate) chain in amounts above 14 mol %, totally inhibits the crystallinity of the polyester. This is not unexpected at all given the large differences in both nature and size between the ES or BS units and the RF units.

**Figure 9:**
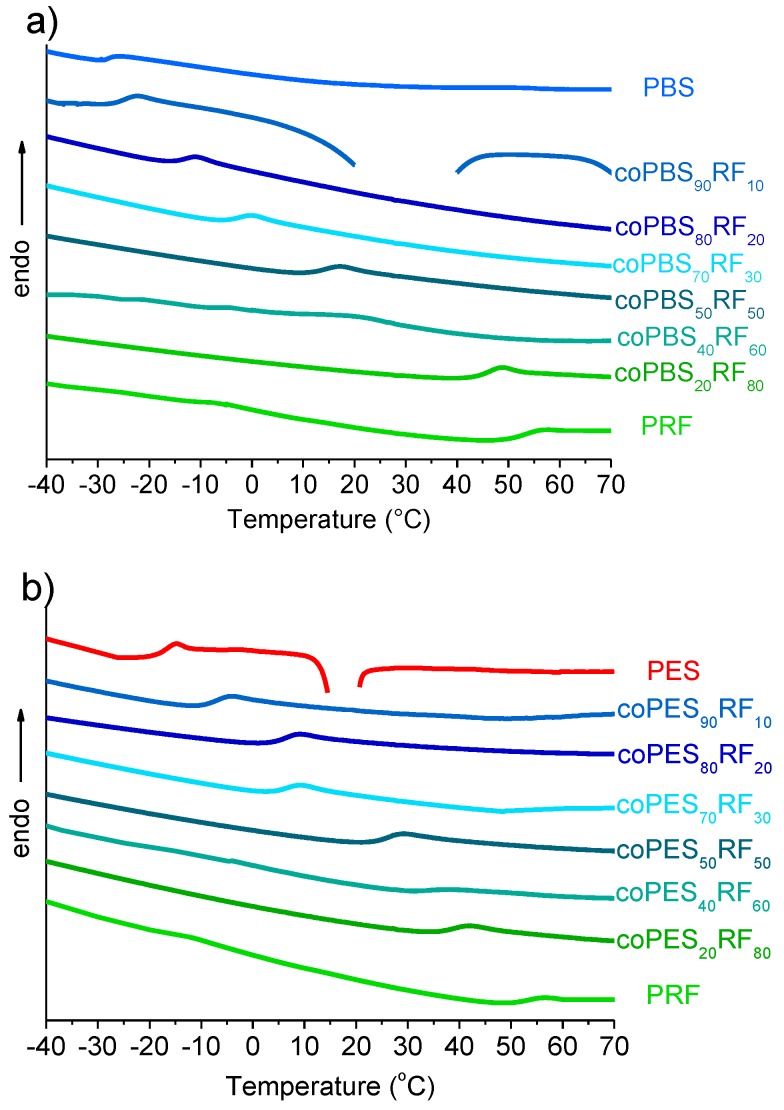
DSC traces showing the *T*_g_ of the two series of resorcinol-containing succinate-furanoatecopolyesters, *co*PBS_x_RF_y_ (**a**) and *co*PES_x_RF_y_ (**b**).

The evaluation of the glass-transition temperature in these copolyesters has deserved particular attention since tuning this parameter by adjusting the content in RF units constitutes one of the main aims of this work. *T*_g_ was measured by DSC from samples that were previously heated at 200 °C to erase their previous history. The thermograms of all the members of the two copolyesters series are comparatively depicted in Figure 9 where *T*_g_ is denoted as a clear positive inflection of the trace recorded at heating, and their numerical values are given in Table 3.

The conclusions that may be drawn from these data, are the following: (a) In both series, *T*_g_ varies with composition monotonically between the values of the two respective parent homopolyesters largely according to what is predicted by both the Fox [33] and the Gordon-Taylor [34] equations with a better fitting for the latter case (see Figure S4 in the SI file). The slight deviations observed between predicted and experimental values could be explained, at least in part, by differences in molecular weight. These results are in full agreement with the random microstructure determined for these copolyesters by NMR and are consistent with the absence of crystallinity that in general is observed in their DSC traces. (b) Incorporation of the RF units entails an increasing of the *T*_g_ with a slope noticeably higher for the *co*PBS_x_RF_y_ series in consistence with the lower *T*_g_ of PBS compared to PES. (c) Differences in *T*_g_ between the two series diminish as the content in RF increases to finally converge in 58 °C which is the *T*_g_ of the PRF homopolyester. 

**Figure 10:**
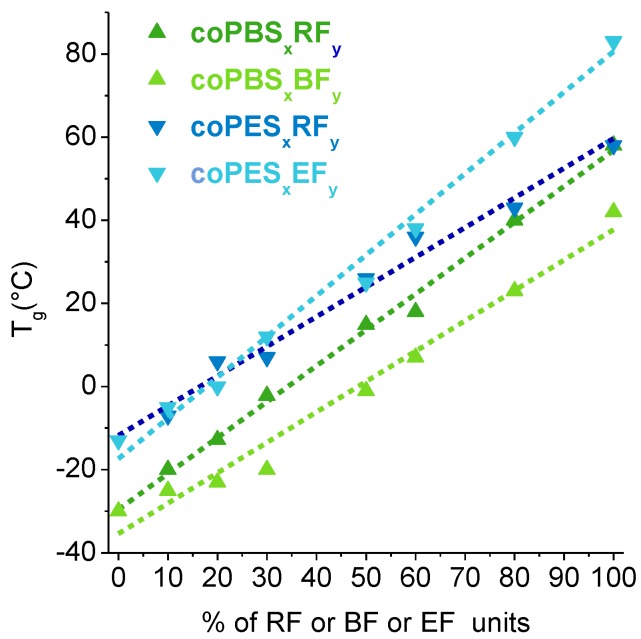
*T*_g_ of furanoate-succinate copolyesters as a function of composition. Data reported by Morales-Huerta et al. [14] for *co*PBS_x_BF_y_ and Terzoupolou et al. [15] for *co*PES_x_EF_y_ are included for comparison.

Random copolyesters of PBS and PES containing furanoate units, *co*PBS_x_BF_y_ and *co*PES_x_EF_y_ respectively, have been described previously and the effect of copolymerization on *T*_g_ has been evaluated for both families [14,15]. In these cases the relatively flexible succinate unit was replaced by the stiffer cyclic furanoate unit while the content in the alkanediol unit (butylene or ethylene) remained constant. As a logical consequence *T*_g_ increased steadily with the content in furanoate units in the two series. The *co*PBS_x_RF_y_ and *co*PES_x_RF_y_ copolyesters studied in this work may be envisaged as deriving from *co*PBS_x_BF_y_ and *co*PES_x_EF_y_ where either the butylene (B) or the ethylene (E) units are replaced by the 1,3-benzenediethoxyethylene (R) units. It is of interest therefore to compare the influence that composition has on *T*_g_ in both types of copolyesters. The *T*_g_ of the two series pairs is plotted against composition in Figure 10 to show the enhancing effect that is exerted by the insertion of aromatic units in the all four cases. Comparison between the two ethylene-based series, i.e. *co*PES_x_EF_y_ and *co*PES_x_RF_y_, shows similar *T*_g_ values in the two series for similar compositions in furanoate units except for very high contents in these units. Conversely, *T*_g_ in the *co*PBS_x_RF_y_ series is invariably higher than in the *co*PBS_x_BF_y_ series with differences becoming larger as copolyesters contain higher amounts of furanoate units. These results reflect the relative stiffness of the diol units that are involved in each case, the 1,3-benzenediethoxyethylene units being intermediate between the ethylene and butylene units.

### 3.5. Biodegradability

**Figure 11:**
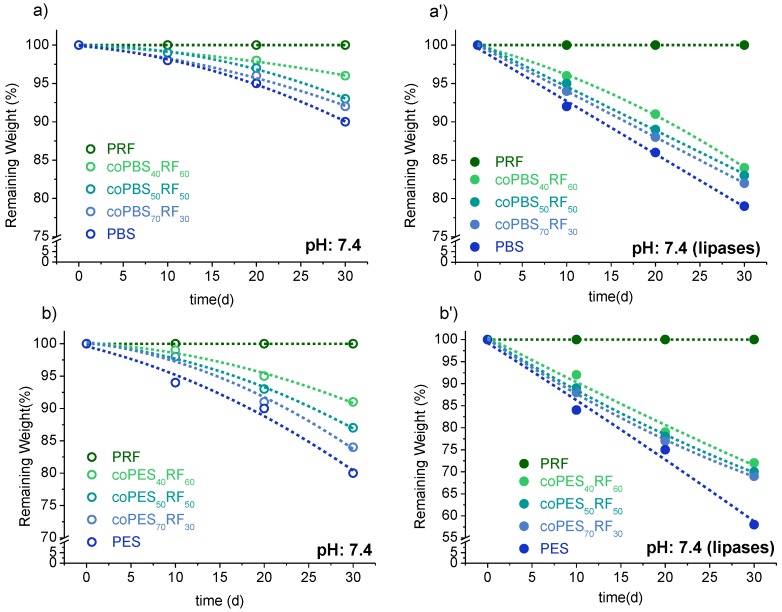
Weight loss of resorcinol-containing succinate-furanoatecopolyesters as a function of incubation time in aqueous buffer at pH 4 and 37 °C (**a**,**b**) and under the same conditions but with lipases added to the incubation medium (**a’**,**b’**).

The biodegradability of the resorcinol-containing succinate-furanoatecopolyesterswas evaluated by following the evolution of sample weight and molecular weight with time in samples with selected compositions that were incubated in aqueous buffer at pH 7.4 and 37 °C with or without lipases added. Sample weight loss data collected for copolyesters containing 30, 50 and 60 mol % for the two series are plotted in Figure 11. No weight change was observed for PRF under any assayed conditions whereas PBS and PES lost approximately 10% and 20% of weight, respectively, when incubated in absence of lipases for 30 days. These amounts became approximately 20–30% greater when lipases were added to the incubation medium revealing the notable susceptibility of these poly(alkylene succinate)sto biodegradation, a fact that is widely known in the related literature [34,35]. The results obtained for *co*PBS_x_RF_y_ and *co*PES_x_RF_y_copolyesters were fully consistent with those obtained for their parent homopolyesters. In both series significant weight losses were observed upon incubation with values steadily increasing with time and being significantly higher when lipases were added to the medium. A similar behavior was displayed by these copolyesters when degradation was followed by measuring the molecular weight by GPC. The *M*_w_-*t* plots, which are accessible in the SI file (Figure S5), revealed decays in molecular weight that were more noticeable in the samples subjected to the enzymes action.

**Figure 12:**
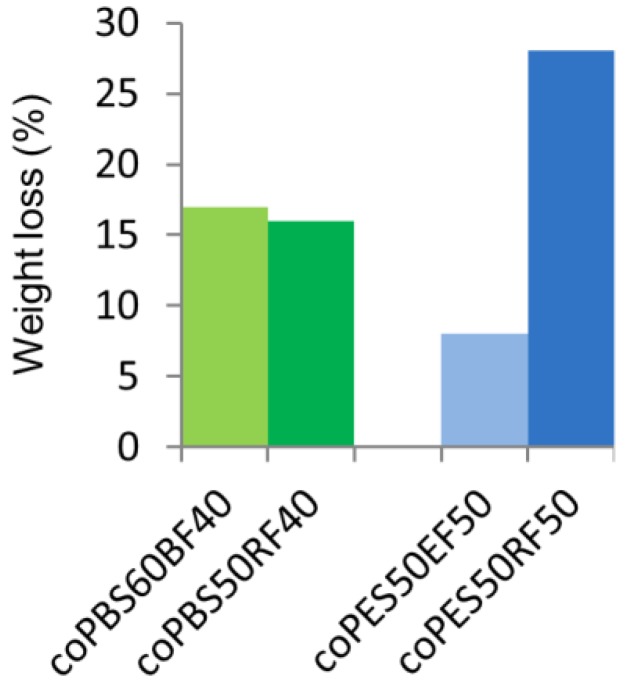
Compared biodegradability (% of weight lost after incubation with lipases) of butylene and ethylene succinate-furanoate copolyesters differing in the content in resorcinol-derived units. Data reported by Morales-Huerta et al. [14] for *co*PBS_60_BF_40_, and Terzoupolou et al. [15] for *co*PES_50_EF_50_ are included for comparison.

A reference of the biodegradation results obtained here for *co*PBS_x_RF_y_ and *co*PES_x_RF_y_ to those reported for the *co*PBS_x_BF_y_ and *co*PES_x_BF_y_ series seems opportune. Comparison had to be however restricted to those particular compositions for which biodegradation data are provided by the different works, which actually are limited to the *co*PBS_40_RF_60_/*co*PBS_40_BF_60_ and *co*PES_50_RF_50_/*co*PES_50_EF_50_ pairs. Biodegradability of these four copolyesters taken as the sample weight loss observed upon incubation with enzymes is compared in the graphic bar depicted in Figure 12. In this plot it is clearly shown that the sensitivity of copolyesters made from BD to enzymatic degradation is scarcely dependent on the substitution of this alkanediol by HER. On the contrary, comparison of PES_50_RF_50_ with PES_50_EF_50_ reveal that the weight lost in the former was about five times than in the latter, which is indicative that *co*PES_x_RF_y_ copolyesters are much more sensitive to biodegradation than their *co*PES_x_EF_y_ analogs containing similar amounts of furanoate units. The rather striking different behavior displayed by butylene and ethylene based copolyesters could be explained taken into account the relative differences in both *T*_g_ and crystallinity between the two series of copolyesters under comparison. 

## 4. Conclusions

Di-*O*-2-(hydroxyethyl) resorcinol, an aromatic diol of bio-based origin, has been used to replace either 1,4-butanediol or ethylene glycol in the synthesis of random copolyesters made of 2,5-furandicarboxylic and succinic acids. These copolyesters are distinguished by being fully sustainable and containing high amounts of aromatic units. The copolyesters are amorphous and display a more-than-good thermal stability with onset temperatures not far from 300 °C. The incorporation of the aromatic units causes an increasing in the *T*_g_ that is more noticeable in the case of the butylene containing copolyesters. The substitution of the alkanediol by resorcinol does not repress the biodegradability of the copolyesters but contrarily it is even enhanced in the case of 1,4-butanediol.

The ROP method has been proven to be an excellent tool for the preparation of these copolyesters. Cyclic oligomers of furandicarboxylate of di-*O*-2-(hydroxyethyl) resorcinol could be successfully prepared by means of the high-dilution condensation method, and copolymerization with oligo(alkylene succinate) cycles proceeded in short time and rendered good yields. The resulting copolyesters had satisfactory molecular weights and their compositions are very similar to those of the feeds used for their synthesis. These copolyesters display slightly yellow-to-brown coloration, an undesirable feature that is known to be common to furanoate-based polymers [36]. The copolymerization approach described in this paper is of interest for modulating the *T*_g_ of poly(alkylene succinate)s without much detriment of their polyester sustainability.

## Figures and Tables

**Table 1:** Cyclic Oligomers Fractions used for ROP.

Fraction	Precursors	Yield (%) ^a^	Composition (% wt) ^b^	Thermal Properties		
				*T*_m_ (°C) ^c^	°*T*_d,5%_ (°C) ^d^	^max^*T*_d_ (°C) ^d^
*c*(BS)_n_	DMS + BD	70	50/40/10	105	266	340/400
*c*(ES)_n_	DMS + EG	70	0/45/55	100	200	300/400
*c*(RF)_n_	FDCA-Cl_2_ + HER	60	28/57/15	158/166/185	263	415

^a^ Yields for the oligomeric after the removal of the linear species (flashed). ^b^ Mixtures of dimer/trimer/tetramer species measured by HPLC. ^c^ Determined by DSC at 10 °C·min^−1^. ^d^ Decomposition temperatures (onset and maximum rate) determined by TGA.

**Table 2:** Resorcinol-containing Succinate-FuranoateCopolyesters Obtained by ROP.

Opolyester	Yield (%)	GPC ^a^		Composition ^b^ x(BS or ES)/y(RF)		Chain Microstructure ^c^				
		*M*_w_ (g·mol^−1^)	*Đ*	Feed	Copolyester	*n*_(B/E)S_	*n*_(B/E)F_	*n*_RS_	*n*_RF_	*B*
PBS	90	50,000	1.9	100/-	100/-	-	-	-	-	-
*co*PBS_90_RF_10_	96	48,000	2.0	90/10	86/14	17.3	1.1	2.8	1.5	0.72
*co*PBS_80_RF_20_	95	46,000	1.8	80/20	78/22	7.2	1.2	3.8	1.4	0.89
*co*PBS_70_RF_30_	90	42,500	1.6	70/30	64/36	4.2	1.3	2.8	1.6	0.90
*co*PBS_50_RF_50_	80	37,200	2.0	50/50	49/51	2.3	1.9	2.1	1.9	0.96
*co*PBS_40_RF_60_	80	36,000	1.7	40/60	37/63	1.8	2.2	1.7	2.5	0.96
*co*PBS_20_RF_80_	85	32,200	1.8	20/80	21/79	1.5	3.2	1.3	3.9	0.92
PES	90	45,000	1.7	100/-	100/-	-	-	-	-	-
*co*PES_90_RF_10_	95	42,500	1.9	90/10	86/14	4.6	1.3	7.3	1.2	1.07
*co*PES_80_RF_20_	92	41,000	1.8	80/20	77/23	3.3	1.4	4.9	1.3	1.17
*co*PES_70_RF_30_	80	39,200	1.5	70/30	76/24	3.0	1.5	4.2	1.3	1.08
*co*PES_50_RF_50_	85	34,500	1.4	50/50	49/51	1.5	3.2	2.3	1.8	1.23
*co*PES_40_RF_60_	90	34,000	1.6	40/60	36/64	1.7	2.5	1.6	2.7	0.98
*co*PES_20_RF_80_	91	31,000	1.8	20/80	19/81	1.3	4.1	1.2	5.6	0.95
PRF	80	28,000	1.9	-/100	-/100	-	-	-	-	-

^a^ Molecular weight and dispersity measured by GPC. ^b^ Composition of the feed and the copolyester. ^c^ Number-average sequence length of the different sequences present in the copolyesters (see text) and randomness (*B*) determined by ^13^C NMR spectra using the equations reported by Devaux [31].

**Table 3:** Thermal Properties of *co*PBS_x_RF_y_ and *co*PES_x_RF_y_ Copolyesters.

Copolyester	TGA ^a^			DSC ^b^		
	°*T*_d_ (°C)	^max^*T*_d_ (°C)	*R*_w_ (%)	*T*_g_ (°C)	*T*_m_ (°C)	∆*H* (J·mol^−1^)
PBS	350	399	3	−30	114	82
*co*PBS_90_RF_10_	336	391	8	−20	100	74
*co*PBS_80_RF_20_	344	398	6	−13	-	-
*co*PBS_70_RF_30_	326	391	9	−2	-	-
*co*PBS_50_RF_50_	336	399	10	15	-	-
*co*PBS_40_RF_60_	331	391	11	18		
*co*PBS_20_RF_80_	345	400	9	40	-	-
PES	310	385	7	−13	100	55
*co*PES_90_RF_10_	323	389	7	−7	-	-
*co*PES_80_RF_20_	335	386	9	6	-	-
*co*PES_70_RF_30_	325	374	9	8	-	-
*co*PES_50_RF_50_	342	390	9	26	-	-
*co*PES_40_RF_60_	340	396	5	36	-	-
*co*PES_20_RF_80_	347	411	11	43	-	-
PRF	321	401	10	58	-	-

^a^ Thermal decomposition temperatures (°*T*_d_ and ^max^*T*_d_) and remaining weight (*R*_w_) determined by TGA. ^b^ Glass-transition temperature and melting temperature and enthalpydetermined by DSC at heating.

## Supplementary Materials

The following are available online at www.mdpi.com/2073-4360/9/12/701/s1.
