# Supplementary material for: Modulating the Tg of Poly(alkylene succinate)s by Inserting Bio-Based Aromatic Units via Ring-Opening Copolymerization
Source: Polymers (Basel). 2017 Dec 12;9(12):701. doi: 10.3390/polym9120701 (PMC6418826; doi:10.3390/polym9120701)
Supplement: Supplementary file 1 [file polymers-09-00701-s001.pdf]

SUPPLEMENTARY INFORMATION

Modulating the T_g of Poly(alkylene succinate)s by Inserting Bio-based Aromatic Units via Ring-Opening Copolymerization

Juan Carlos Morales-Huerta, Antxon Martínez de Ilarduya and Sebastián Muñoz-Guerra

Departament d'Enginyeria Química, Universitat Politècnica de Catalunya, ETSEIB, Diagonal 647, 08028 Barcelona, Spain

Correspondence: sebastian.munoz@upc.edu

Contents:

Figure S1. ^{1}H NMR of *co*PBS$_x$RF$_y$ and *co*PES$_x$RF$_y$.

Figure S2. ^{13}C NMR of *co*PBS$_x$RF$_y$ and *co*PES$_x$RF$_y$.

Figure S3. ^{13}C NMR spectra of *co*PES$_x$RF$_y$ copolyesters in the regions of the carbonyls and aromatic carbons.

Figure S4. Comparison of predicted (dashed lines) and experimental (symbols) values of T_g for the copolyesters synthesized in this work. a) Fox equation. B) Gordon-Taylor equation.

Figure S5. Molecular weight reduction of resorcinol-containing copolyesters as a function of incubation time in aqueous buffer at pH 4 and 37 ºC (a and b) and under the same conditions but with lipases added to the incubation medium (a' and b').

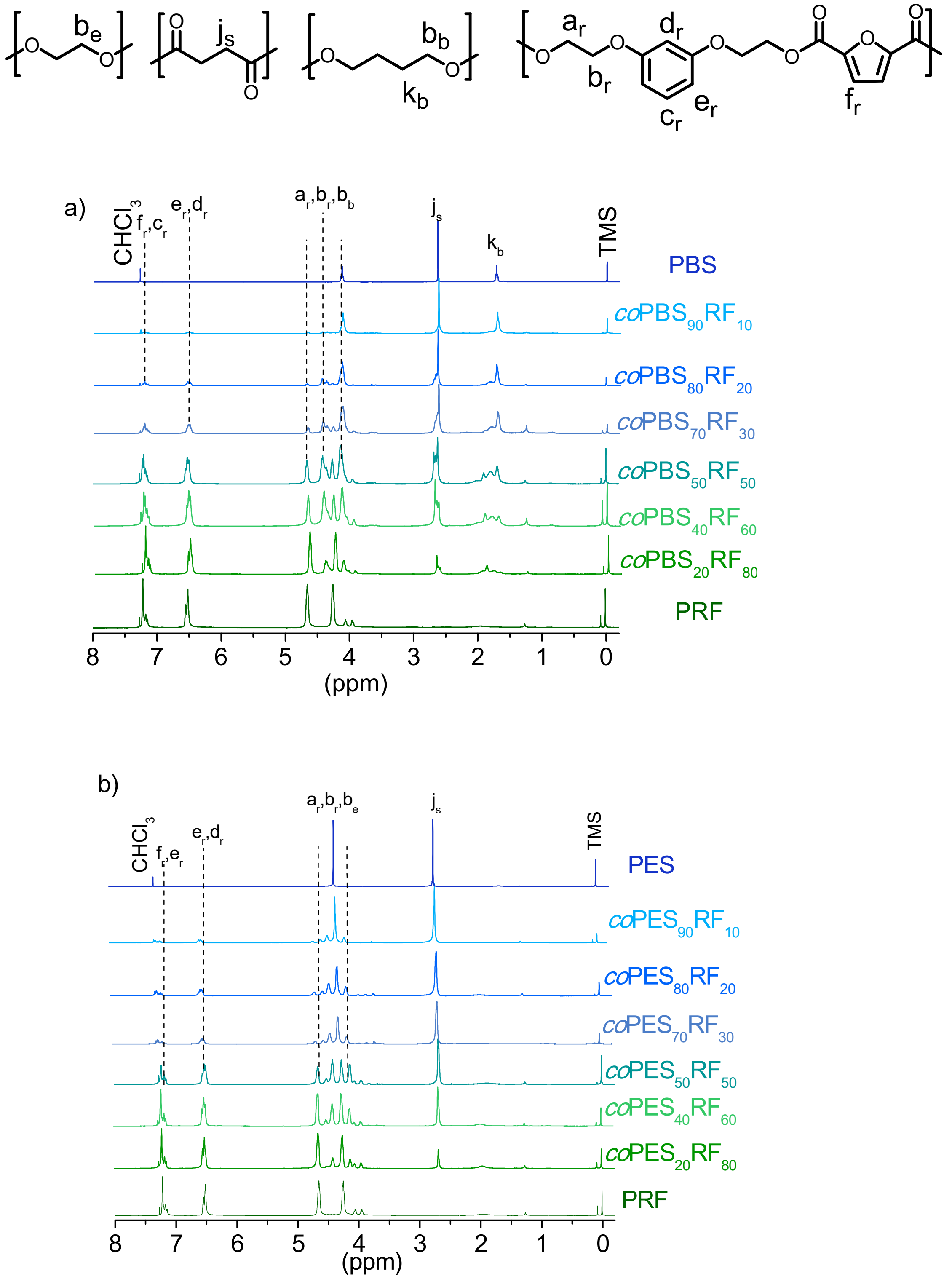

b_e
j_s
b_b
k_b
a_r
d_r
b_r
c_r
e_r
f_r
a)
CHCl3
f_r,c_r
e_r,d_r
a_r,b_r,b_b
j_s
k_b
TMS
PBS
coPBS90RF10
coPBS80RF20
coPBS70RF30
coPBS50RF50
coPBS40RF60
coPBS20RF80
PRF
8 7 6 5 4 3 2 1 0
(ppm)
b)
CHCl3
f_r,e_r
e_r,d_r
a_r,b_r,b_e
j_s
TMS
PES
coPES90RF10
coPES80RF20
coPES70RF30
coPES50RF50
coPES40RF60
coPES20RF80
PRF
8 7 6 5 4 3 2 1 0
(ppm)

Figure S1. ^{1}H NMR of *co*PBS$_x$RF$_y$ and *co*PES$_x$RF$_y$.

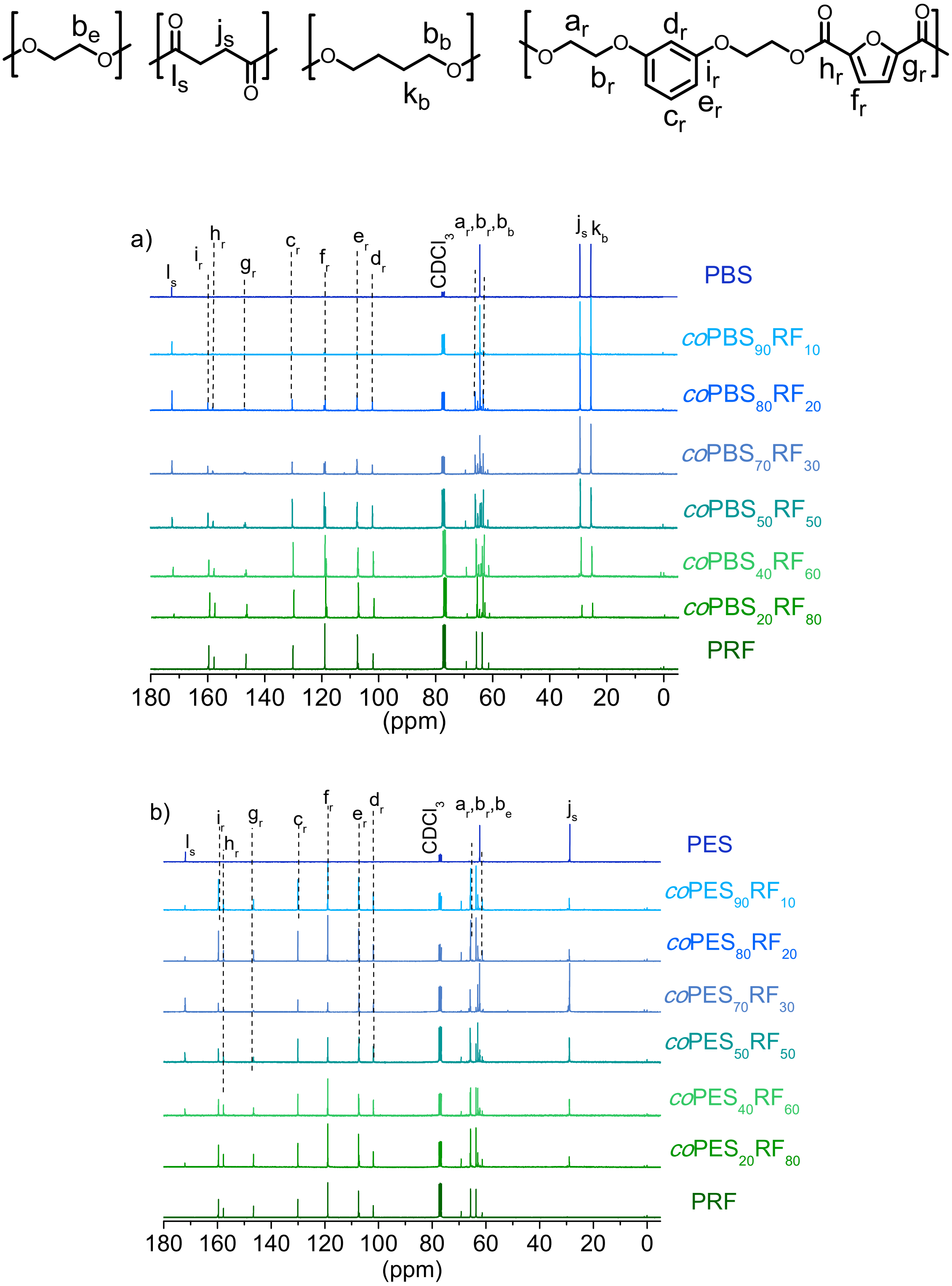

Figure S2. ^{13}C NMR of *co*PBS$_x$RF$_y$ and *co*PES$_x$RF$_y$.

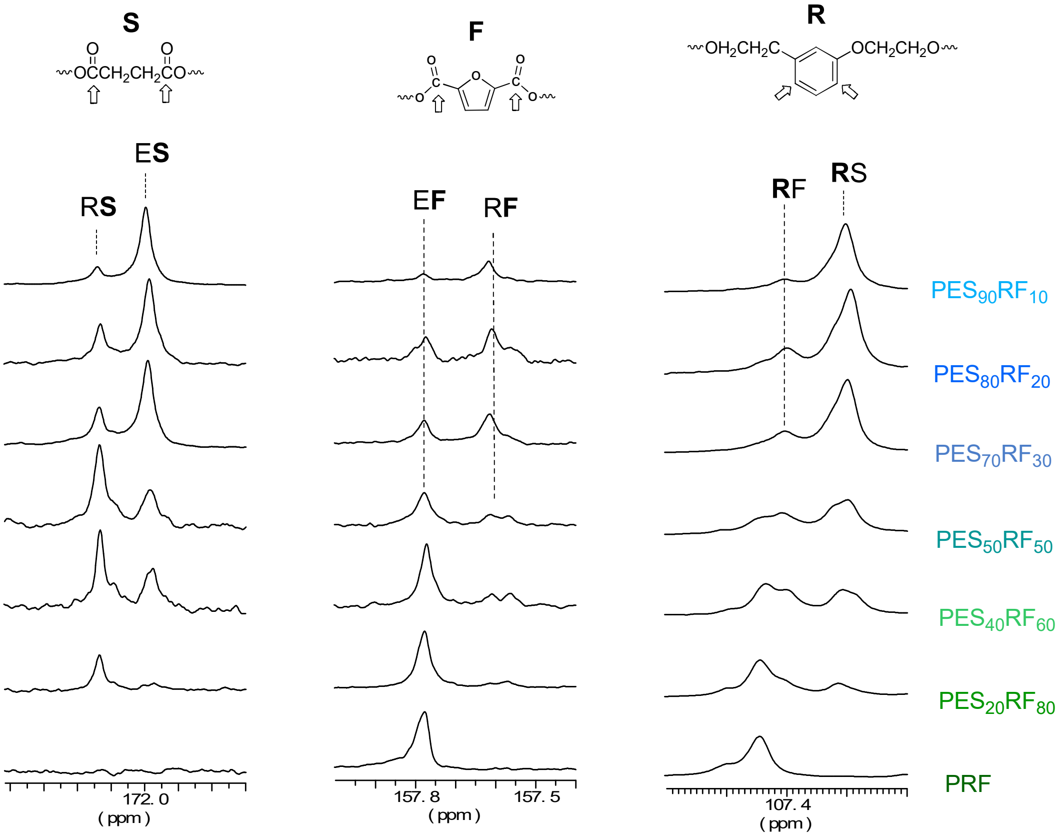

Figure S3. ^{13}C NMR spectra of *co*PES_xRF_y copolyesters in the regions of the carbonyls and aromatic carbons.

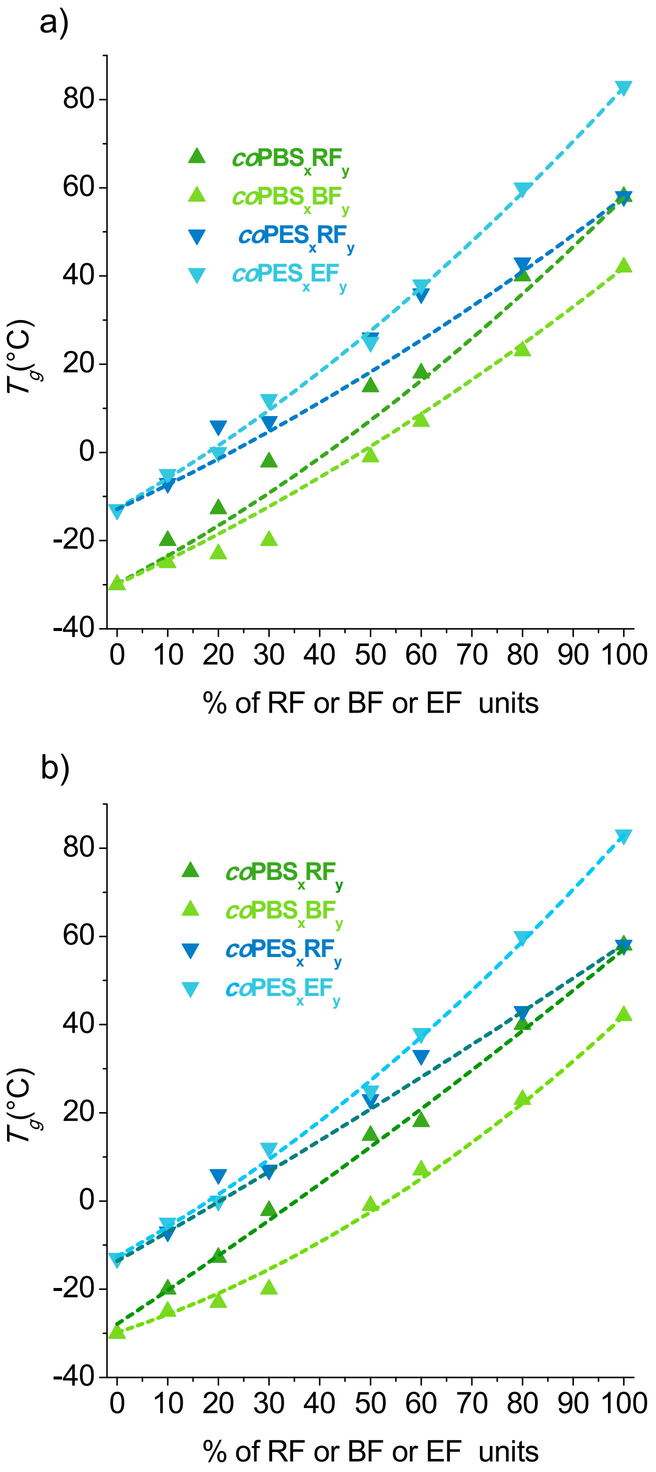

PE	ΔCp ($J \cdot g^{-1} \cdot {}^{\circ}C^{-1}$)
PBS	0.618
PBF	0.364
PES	0.645
PEF	0.274
PRF	0.469

Figure S4. Comparison of predicted (dashed lines) and experimental (symbols) values of T_g for the copolyesters synthesized in this work. a) Fox equation. b) Gordon-Taylor equation. Inset: Heat capacity values measured by DSC for each homopolyester for K determination.

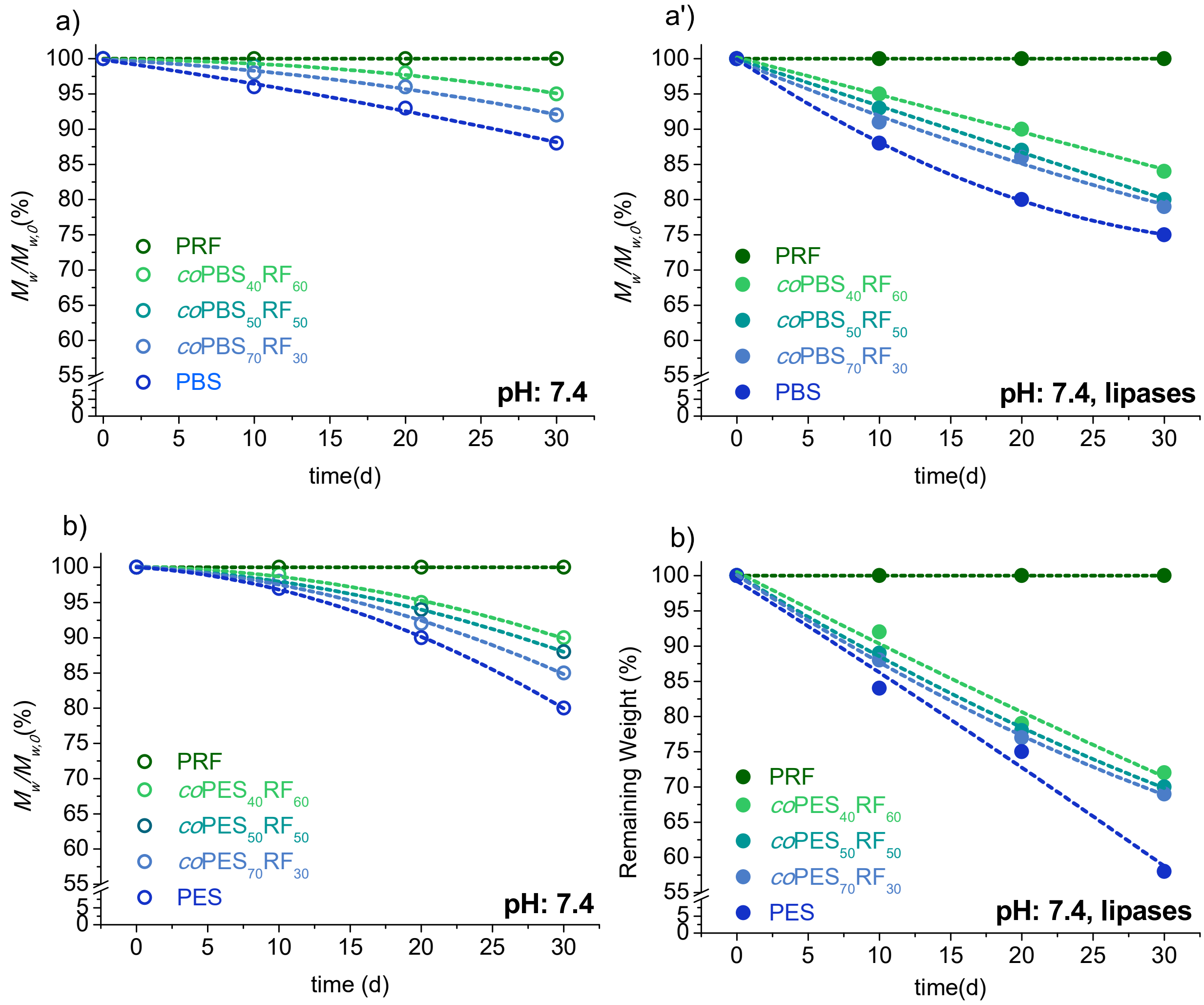

Figure S5. Molecular weight reduction of resorcinol-containing succinate-furanoate copolyesters as a function of incubation time in aqueous buffer at pH 4 and 37 ºC (a and b) and under the same conditions but with lipases added to the incubation medium (a’ and b’).
